# Astragalosidic Acid: A New Water-Soluble Derivative of Astragaloside IV Prepared Using Remarkably Simple TEMPO-Mediated Oxidation

**DOI:** 10.3390/molecules22081275

**Published:** 2017-07-31

**Authors:** Lin-Sen Qing, Shu-Lin Peng, Jian Liang, Li-Sheng Ding

**Affiliations:** Chengdu Institute of Biology, Chinese Academy of Sciences, Chengdu 610041, China; qingls@cib.ac.cn (L.-S.Q.); pengsl@cib.ac.cn (S.-L.P.); liangjian@cib.ac.cn (J.L.)

**Keywords:** astragalosidic acid, astragaloside IV, water-soluble derivative, TEMPO-mediated oxidation, cardioprotective activity

## Abstract

There is an urgent need for a water-soluble derivative of astragaloside IV for drug R&D. In the present study, a remarkably simple method for the preparation of such a water-soluble derivative of astragaloside IV has been developed. This protocol involves oxidative 2,2,6,6-tetramethylpiperidine-1-oxyl free radical (TEMPO)-mediated transformation of astragaloside IV to its carboxylic acid derivative, which is a new compound named astragalosidic acid. The structure of astragalosidic acid was elucidated by means of spectroscopic analysis. Its cardioprotective activity was investigated using an in vitro model of cardiomyocyte damage induced by hypoxia/reoxygenation in H9c2 cells. The oxidative TEMPO-mediated transformation proposed in the present study could be applied to other natural saponins, offering an effective and convenient way to develop a new compound with greatly improved structure-based druggability.

## 1. Introduction

Astragali Radix is a well-known herbal drug in traditional Chinese medicine which has been used to treat numerous diseases for hundreds of years. To date, more than 200 kinds of traditional Chinese medicine patent prescription containing Astragali Radix extract have been approved by the China Food and Drug Administration. Astragaloside IV (3-*O*-β-d-xylopyranosyl-6-*O*-β-d-glucopyranosyl-cycloastragenol), an active substance of Astragali Radix, has been used as a chemical marker for quality control of Astragali Radix and its preparation in the Chinese Pharmacopoeia [1]. Despite its well-documented pharmacological effects [2,3], the druggability of astragaloside IV, related to its characteristics of solubility, dissolution, and gastrointestinal permeability, has always attracted research interest. Poor water solubility and poor dissolution in the gastrointestinal fluids of astragaloside IV is a limiting factor to in vivo bioavailability [4,5]. Therefore, there is an immediate need for a water-soluble derivative of astragaloside IV using a low-cost preparation method in drug R&D.

Due to the structural complexity of astragaloside IV (it has a number of potential chemical reaction sites), so far there has been no feasible reported method for preparing a water-soluble derivative. Salt formation is the most common and effective way to improve druggability, increasing solubility and dissolution rates of the pharmaceutical compound [6]. Among a variety of chemical approaches, 2,2,6,6-tetramethylpiperidine-1-oxyl free radical (TEMPO) is a classic nitroxide radical, which can selectively oxidize the primary alcohol to carbohydrate [7,8,9]. In this work, a new water-soluble derivative of astragaloside IV, compound **1**, was prepared by remarkably simple TEMPO-mediated oxidation, offering a promising option for natural drug research. Herein, we describe the preparation, structural elucidation, and in vitro cardioprotective activity of this new compound.

## 2. Results and Discussion

### 2.1. Preparation of Compound ***1***

**Figure 1:**
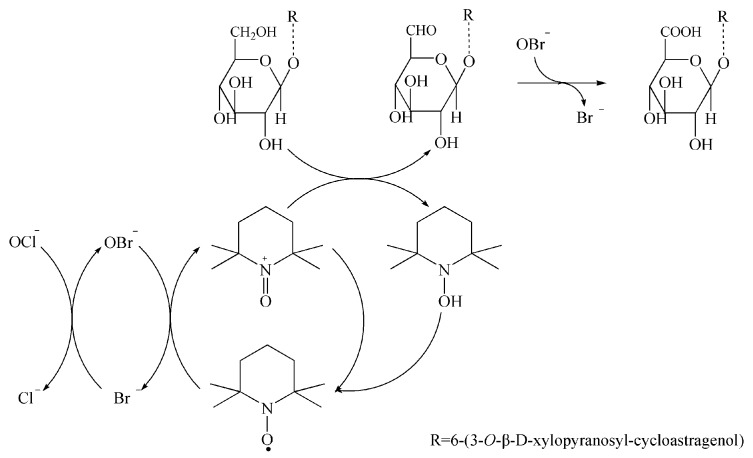
Proposed reaction mechanism for the formation of compound **1**.

A total of 200 mg astragaloside IV was added in to 200 mL water containing 100 mg NaBr, 40 mg TEMPO, and 1 mL NaClO. The reaction mixture was stirred rapidly in open air at 20 ± 0.5 °C and monitored by thin-layer chromatography (TLC) until no starting material remained. After reacting for 2 h, the mixture was filtered, and the oxidation was terminated by adjusting the pH to 2. The reaction mixture was concentrated to about 10 mL, filtered, and then washed by 2 mL acid water to obtain compound **1** (172 mg) with a yield of 84.5%. The reaction pathway is depicted in Figure 1.

In recent decades, catalytic oxidation of carbohydrates using TEMPO (a stable nitroxyl radical) has become one of the most promising procedures for converting sugars into corresponding uronic acids. The method is very suitable for selective oxidation of primary alcohol groups into aldehydes and/or carboxylic acid groups with high selectivity, and high reaction rates and yield [7,8,9,10]. The present study is the first report of selective oxidation of saponin through the primary alcohols on its sugar chains, which can be applied to other saponins such as protodioscin, ginsenoside Rg1, and notoginsenoside R1.

### 2.2. Structure Elucidation of Compound ***1***

Compound **1** was obtained as a white powder with ${\left[\alpha \right]}_{D}^{20}$ + 8.4° (c, 1.0, MeOH), and IR (KBr) γ_max_ 3417, 2968, 1732, 1621, 1378, 1156, 1066, and 1045 cm^−1^. Its molecular formula was established as C_41_H_66_O_15_ by HR-ESI-MS (*m*/*z* 821.4294 [M + Na]^+^, calcd. for C_41_H_66_NaO_15_: 821.4294), requiring 9° of unsaturation. The ESI-MS fragments were *m*/*z* 665, 621 [M + H]^+^. The TLC *R*_f_ value was 0.3 (CH_2_Cl_2_–MeOH–H_2_O–CH_3_COOH, 10:4:0.5:0.01). The solubilities of compound **1** and its sodium salt were 0.169 mg/mL and 8.35 mg/mL in pure water at 30 °C, respectively.

**Figure 2:**
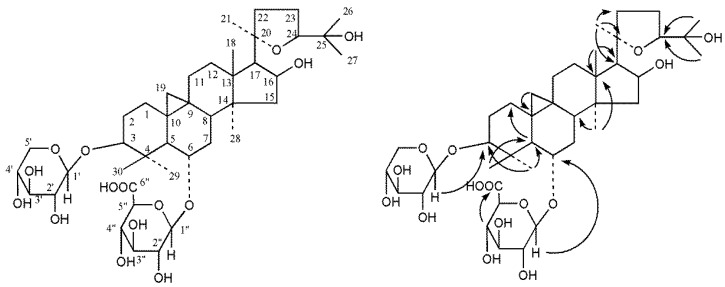
Structure and key HMBC correlations of compound **1**.

The ^1^H-NMR spectrum showed two doublets upfield of δ 0.16 (1H, d, *J* = 4.2 Hz), and 0.54 (1H, d, *J* = 4.2 Hz), which was characteristic of an AB splitting system belonging to the methylene protons of the cyclopropane ring. In addition, there were seven methyl signals at δ 1.96 (H-29), 1.53 (H-27), 1.35 (H-18), 1.29 (H-30), 1.26 (H-26), 1.24 (H-21), and 0.96 (H-28) in the ^1^H-NMR spectrum. These data, together with the ^13^C-NMR data, could classify **1** as a cycloartane triterpenoid. On comparison of the NMR data for astragaloside IV, the aglycone of **1** could be determined as cycloastragenol. The ^1^H and ^13^C-NMR spectra showed two anomeric signals at δ_H_ 4.80 (1H, d, *J* = 7.2 Hz), 5.02 (1H, d, *J* = 7.2 Hz), and δ_C_ 107.4 and 105.4, suggesting that there were two sugar moieties in **1**. Further, the carbohydrates could easily be identified as β-d-xylose and β-d-glucuronic acid by ^1^H-NMR data δ_H_ 4.80 (1H, d, *J* = 7.2 Hz), 5.02 (1H, d, *J* = 7.2 Hz), negative mode ESI-MS fragments at *m*/*z* 665 [M − H − 132]^−^ and 621 [M − H − 176]^−^, and TLC analysis of acidic hydrolysis of **1**. The attachment sites of the carbohydrates in **1** were determined as C-3 and C-6 by glycosylation at δ 88.2 and 79.0 into C-6 (δ_C_ 79.0). The location of sugar was confirmed by ^1^H detected heteronuclear multiple bond correlation (HMBC) analysis as shown in Figure 2. Furthermore, the NMR data of **1** were very similar to those of astragaloside IV [11]. The difference between the two compounds was the sugar moiety attached to C-6. The ^1^H and ^13^C-NMR, DEPT (θ = 90°), DEPT (θ = 135°), ^1^H-^1^H COSY, HSQC, HMBC spectra of compound **1** were available in Supplementary Materials. Hence, the structure of **1** was elucidated as (3β,6α,16β,20*R*,24*S*)-20,24-epoxy-16,25-dihydroxy-3-(β-d-xylopyranosyloxy)-9,19-cyclolanostan-6-yl β-d-glucuronopyranoside (Figure 2) and named astragalosidic acid. The ^13^C-NMR data of compound **1** are listed in Table 1.

### 2.3. Investigation of Cardioprotective Activity In Vitro

**Figure 3:**
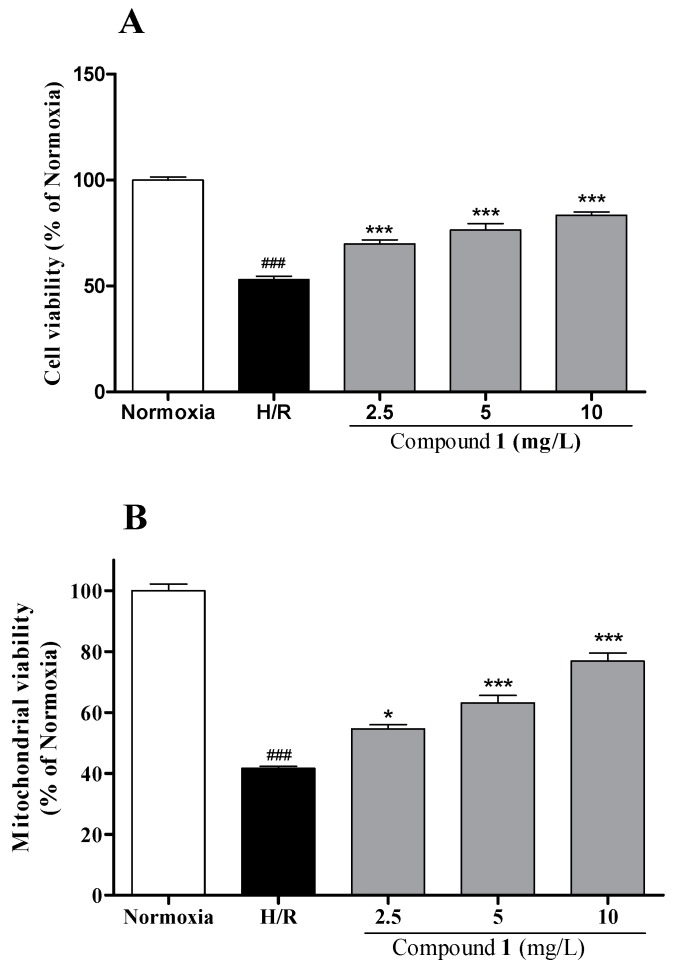
Effects of compound **1** on hypoxia/reoxygenation (H/R)-induced injury in H9c2 cells. (**A**) Cell viability was determined by 3-(4,5-dimethylthiazol-2-yl)-2,5-diphenyltetrazolium bromide (MTT) assay. Cell viability of normal group was considered as 100%; (**B**) Mitochondrial viability was determined by florescent staining. Data represent mean ± SEM. The results were reproduced by six independent experiments. ^###^ indicates *p* < 0.001 for the H/R group vs. normoxia group; * indicates *p* < 0.05, *** indicates *p* < 0.001 for each treatment group vs. the H/R group.

**Figure 4:**
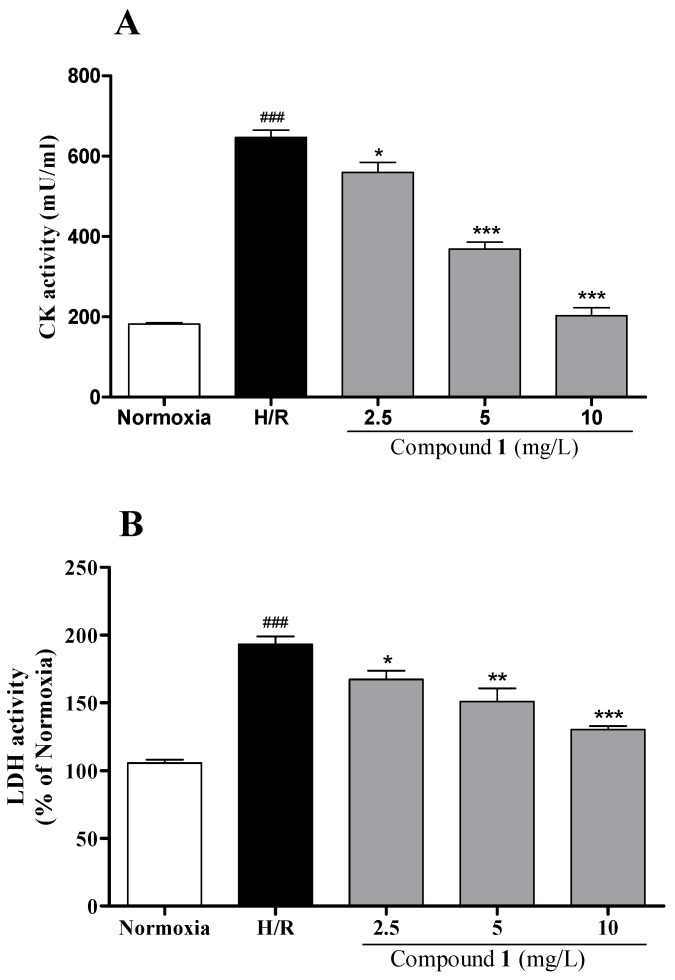
Effects of compound **1** on myocardial enzymes markers in H9c2 cells subjected to H/R. (**A**) Lactate dehydrogenase (LDH); (**B**) Creatine kinase (CK). Data represents mean ± SEM. Myocardial enzymes markers were assessed by a commercial LDH and CK activity assay kit. The results were reproduced by six independent experiments. ^###^ indicates *p* < 0.001 for the H/R group vs. the normoxia group; * indicates *p* < 0.05, ** indicates *p* < 0.01, *** indicates *p* < 0.001 for each treatment group vs. H/R group.

To examine the potential bioactivity of compound **1**, we employed a well-established in vitro cardiomyocyte injury model that was induced by hypoxia/reoxygenation (H/R). As shown in Figure 3, H/R induction significantly reduced the cell viability and mitochondrial viability in H9c2 cells compared with the normoxia group (*p* < 0.001, *p* < 0.001, respectively). Compound **1** at 2.5, 5 and 10 mg/L significantly increased cell viability and mitochondrial viability in a dose-dependent manner, suggesting its protective effect against the H/R-induced cardiomyocyte death. For the myocardial injury detection, compound **1** was investigated by cytosolic creatine kinase (CK) and lactate dehydrogenase (LDH) activity in H9c2 cells. H/R significantly increased LDH release and CK content compared with the normoxia group (*p* < 0.001, *p* < 0.001, respectively). Our results in Figure 4 indicated that compound **1** significantly inhibited the rise of myocardial enzyme markers induced by H/R in a dose-dependent manner.

## 3. Materials and Methods

### 3.1. Apparatus and Materials

Astragaloside IV was prepared in-house from Astragali Radix with structure characterization by multiple spectroscopic analysis (UV, IR, HR-ESI-MS, ^1^H-NMR, and ^13^C-NMR) and comparison with the literature. Bruker AVANCE 600 NMR (Bruker, Zurich, Switzerland) and MicrOTOF QII HR-MS (Bruker Daltonik, Germany) were applied for structure elucidation. TLC plates were prepared in-house with silica gel G and CMC-Na. Milli-Q water (Millipore Corp., Bedford, MA, USA) was used throughout the work. Other chemicals and solvents were of analytical reagent grade. Fetal bovine serum was purchased from Sigma-Aldrich (St. Louis, MO, USA). The rat H9c2 cardiomyocyte cell line was purchased from the American Type Culture Collection (CRL1446, ATCC, Manassas, VA, USA). Dulbecco’s Modified Eagle Medium (Gibco, Langley, OK, USA) and Billups-Rotenberg modular incubator chamber (Billups-Rotenberg, Del Mar, CA, USA) were used for the cells grown. The UV absorbance was determined using the Infinite M200pro microplate reader (Tecan, Mannedorf, Switzerland). The fluorescence was also detected using the Infinite M200pro microplate reader. Mitochondrial viability was evaluated by the mitochondrial viability assay kit (Abcam, Cambridge, UK) according to the manual. Activities of CK and LDH were detected by the commercial assay kits (Biovision, Milpitas, CA, USA) according to the manufacturer’s recommendations.

### 3.2. Acid Hydrolysis of Compound ***1***

Compound **1** (10 mg) was heated with 1.5 M HCl-CH_3_OH (20 mL) under reflux for 8 h. The reaction mixture was subjected to silica gel TLC, together with the standard samples, using CHCl_3_-CH_3_OH-H_2_O-CH_3_COOH (5:6:1:1) as the developing solvent and using *O*-phthalic acid-aniline as the detection reagent. Xylose (*R*_f_ 0.86), and glycuronic acid (*R*_f_ 0.23) were detected.

### 3.3. Bioactivity Evaluation

#### 3.3.1. Cell Culture and In Vitro Cardiomycyte Injury Model Induced by Hypoxia/Reoxygenation (H/R)

Cells were grown in Dulbecco’s Modified Eagle Medium (DMEM) supplemented with 10% fetal bovine serum (FBS) and 1% *v*/*v* penicillin/streptomycin at 37 °C in a 5% CO_2_ humidified atmosphere. Compound **1** was dissolved in culture medium to the indicated final concentrations (2.5, 5 and 10 mg/L) before treatment. Cells were exposed to compound **1** for 30 min, and then subjected to H/R as described by us previously [12]. Briefly, medium was replaced with Krebs–Ringer Bicarbonate (KRB) buffer (115 mM NaCl, 4.7 mM KCl, 2.5 mM CaCl_2_, 24 mM NaHCO_3_, 1.2 mM KH_2_PO_4_, 1.2 mM MgSO_4_, 10 mM Hepes and 0.01% bovine serum albumin), placed in a Billups–Rotenberg modular incubator chamber saturated with 99.99% nitrogen. After 3 h of hypoxia, KRB buffer was replaced with DMEM with 10% FBS under 21% O_2_ for 3 h of reoxygenation.

#### 3.3.2. Cell and Mitochondrial Viability Assay

After treatments, cell viability was determined by 3-(4,5-dimethylthiazol-2-yl)-2,5-diphenyltetrazolium bromide (MTT) assay. The absorbance was detected at 570/650 nm on an Infinite M200pro microplate reader. Mitochondrial viability was evaluated by the mitochondrial viability assay kit according to the manual. Fluorescence was detected at 590 nm with an excitation wavelength of 550 nm by the Infinite M200pro microplate reader.

#### 3.3.3. Creatine Kinase and Lactate Dehydrogenase Activity Detection

The activity of CK and LDH were detected by the commercial assay kits according to the manufacturer’s recommendations.

## 4. Conclusions

In conclusion, the present study reported the preparation, structure elucidation, and cardioprotective activity of astragalosidic acid, a new carboxylic acid derivative of astragaloside IV oxidized by TEMPO-mediated transformation. The results showed that astragaloside acid was a new compound with great potential for new drug R&D. This is the first report of selective oxidation of saponin through the primary alcohols on its sugar chains. Furthermore, the methodology proposed in the present study could be applied to other natural saponins, offering an effective and convenient way to develop a new compound with greatly improved structure-based druggability.

## Figures and Tables

**Table 1:** ^13^C-NMR data (150 MHz) of compound **1** in C_5_D_5_N (δ in ppm).

Position	δ_C_	DEPT	Position	δ_C_	DEPT
1	28.7	CH_2_	23	26.2	CH_2_
2	30.0	CH_2_	24	81.4	CH
3	88.2	CH	25	71.1	C
4	42.4	C	26	28.4	CH_3_
5	52.2	CH	27	28.5	CH_3_
6	79.0	CH	28	19.6	CH_3_
7	34.6	CH_2_	29	28.3	CH_3_
8	45.4	CH	30	16.6	CH_3_
9	20.8	C			
10	27.9	C	d-xylosyl		
11	25.9	CH_2_	1′	107.4	CH
12	33.2	CH_2_	2′	75.4	CH
13	44.8	C	3′	78.3	CH
14	45.9	C	4′	71.0	CH
15	46.0	CH_2_	5′	66.8	CH_2_
16	73.2	CH	d-glucuronyl		
17	58.0	CH	1″	105.4	CH
18	20.9	CH_3_	2″	75.3	CH
19	32.0	CH_2_	3″	78.2	CH
20	87.0	C	4″	73.0	CH
21	26.9	CH_3_	5″	77.1	CH
22	34.6	CH_2_	6″	172.7	C

## Supplementary Materials

Supplementary Materials are available online. ^1^H and ^13^C-NMR, DEPT (θ = 90°), DEPT (θ = 135°), ^1^H-^1^H COSY, HSQC, HMBC spectra of compound **1** are available.
