# Supplementary material for: Astragalosidic Acid: A New Water-Soluble Derivative of Astragaloside IV Prepared Using Remarkably Simple TEMPO-Mediated Oxidation
Source: Molecules. 2017 Jul 31;22(8):1275. doi: 10.3390/molecules22081275 (PMC6152088; doi:10.3390/molecules22081275)
Supplement: Supplementary file 1 [file molecules-22-01275-s001.pdf]

Supplementary data

Supplementary data (^{1}H, ^{13}C, DEPT (θ = 90 °), DEPT (θ = 135 °), H-H COSY, HMBC and HSQC spectra for compound **1**) associated with this article can be found as following:

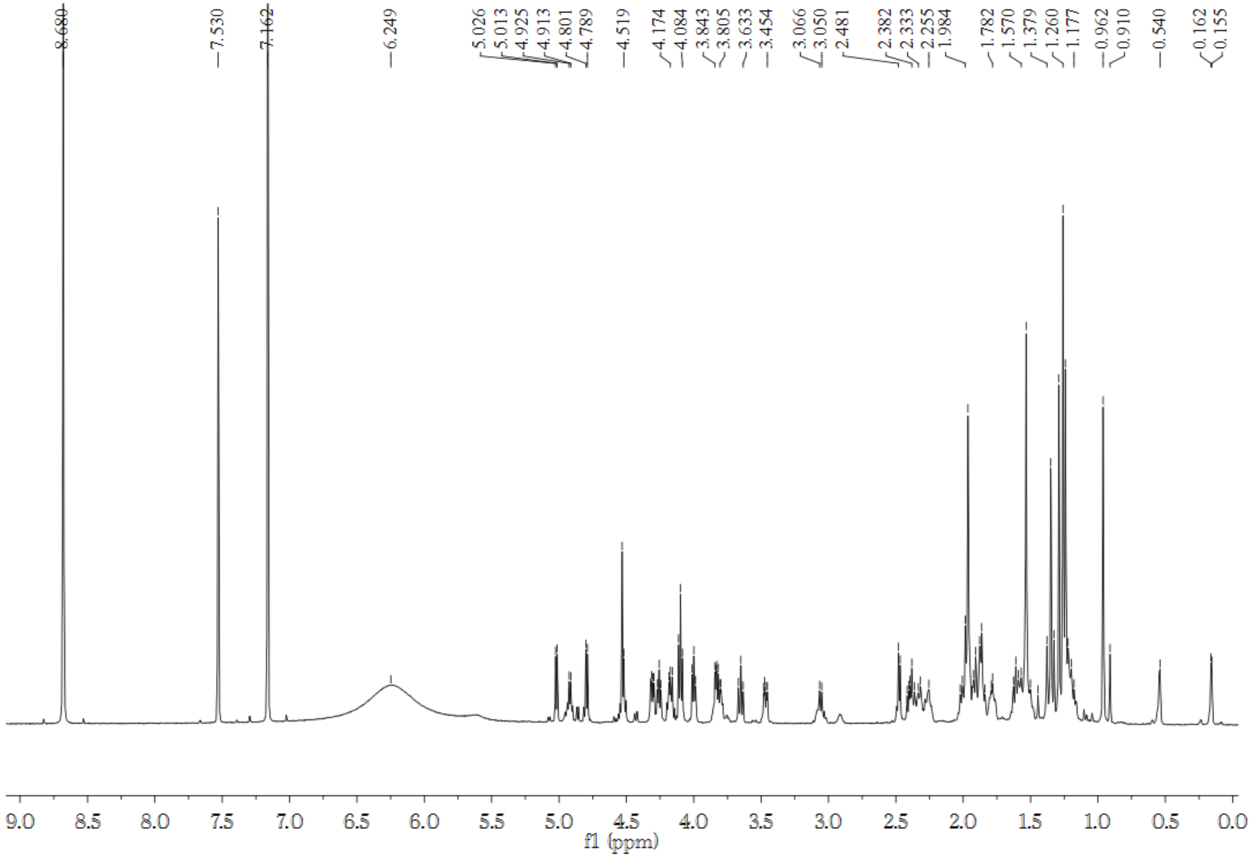

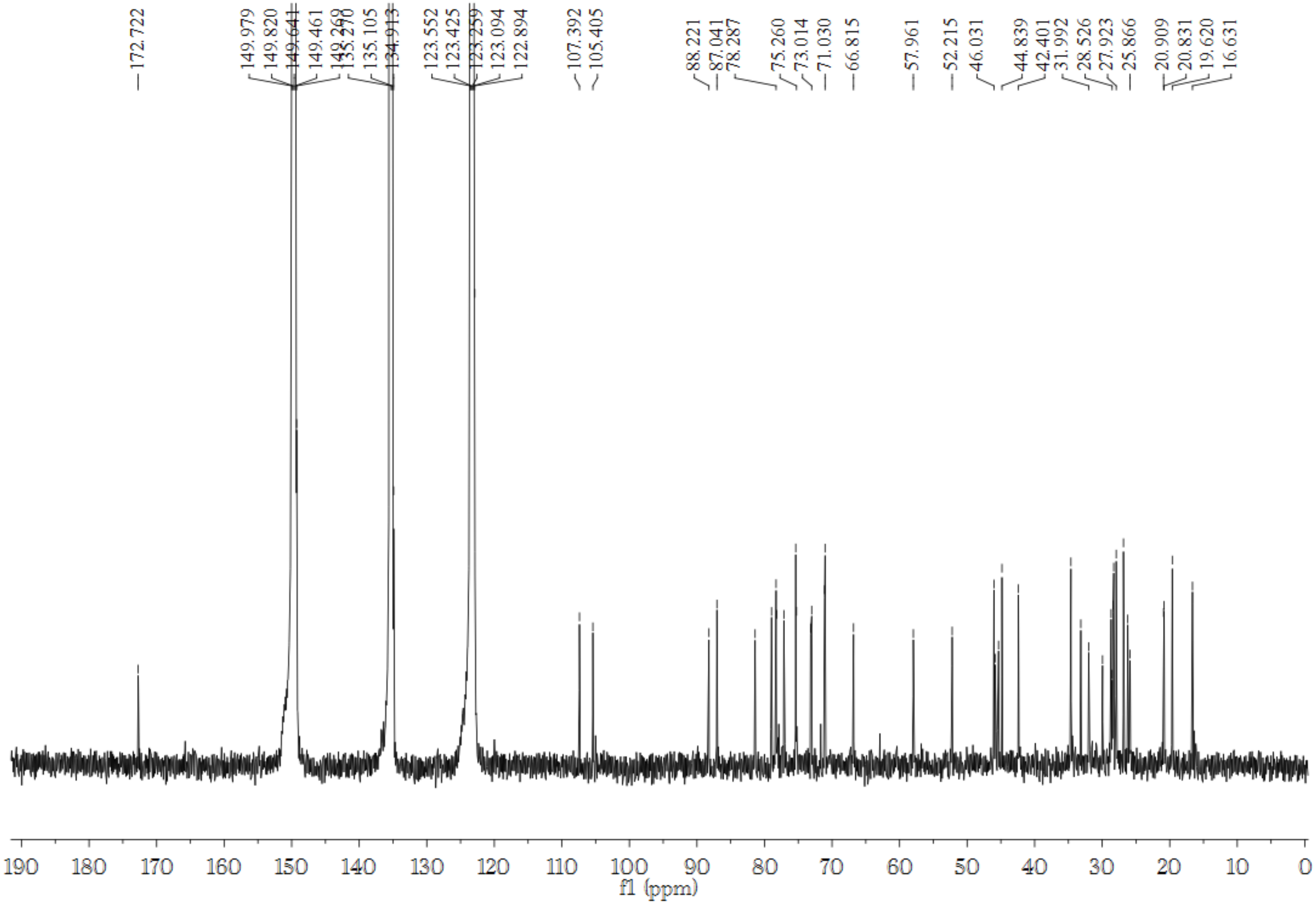
172.722
149.979
149.820
149.641
149.461
149.269
135.270
135.105
134.913
123.552
123.425
123.259
123.094
122.894
107.392
105.405
88.221
87.041
78.287
75.260
73.014
71.030
66.815
57.961
52.215
46.031
44.839
42.401
31.992
28.526
27.923
25.866
20.909
20.831
19.620
16.631
190 180 170 160 150 140 130 120 110 100 90 80 70 60 50 40 30 20 10 0
f1 (ppm)

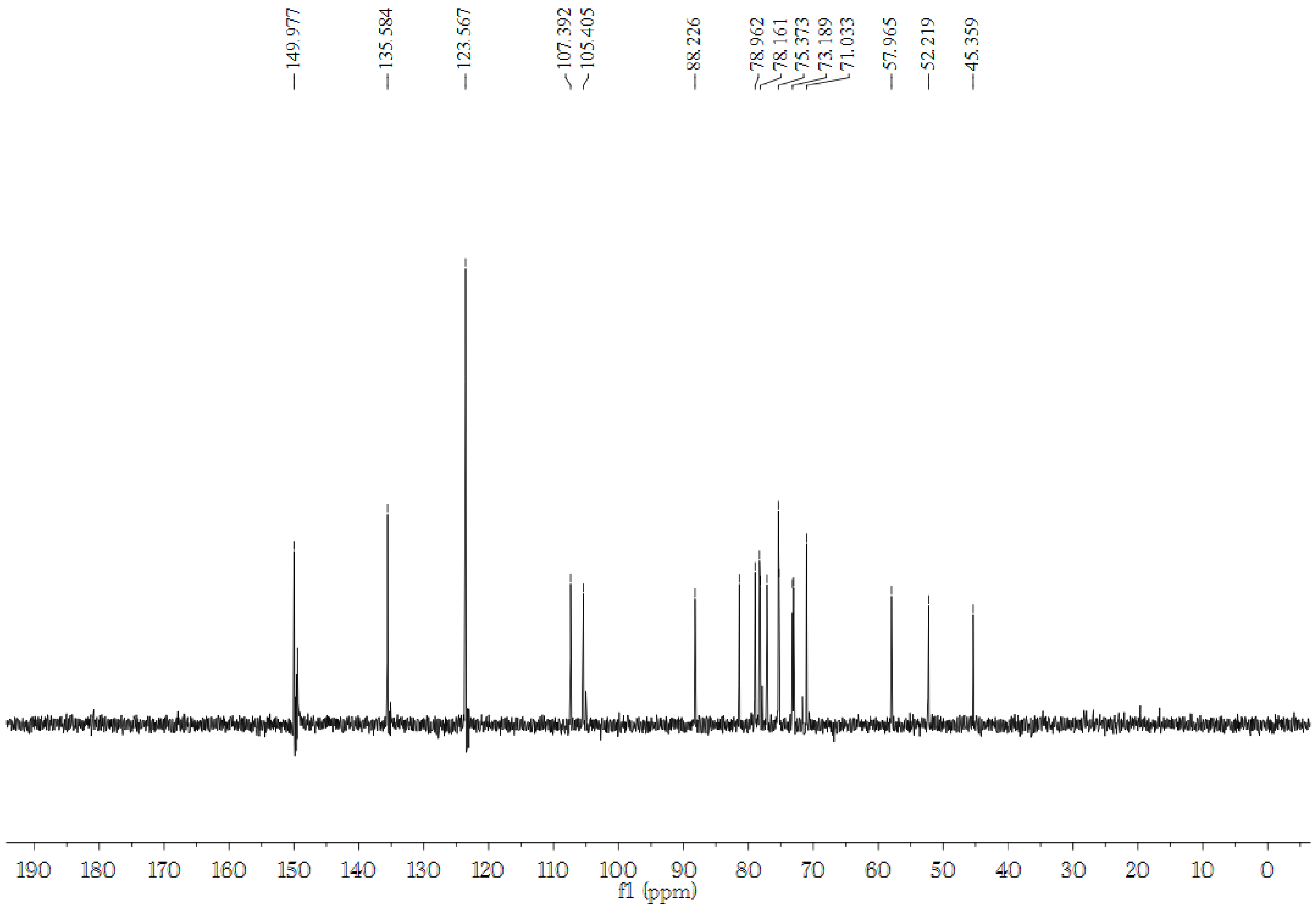

149.977
135.584
123.567
107.392
105.405
88.226
78.962
78.161
75.373
73.189
71.033
57.965
52.219
45.359
190 180 170 160 150 140 130 120 110 100 90 80 70 60 50 40 30 20 10 0
f1 (ppm)

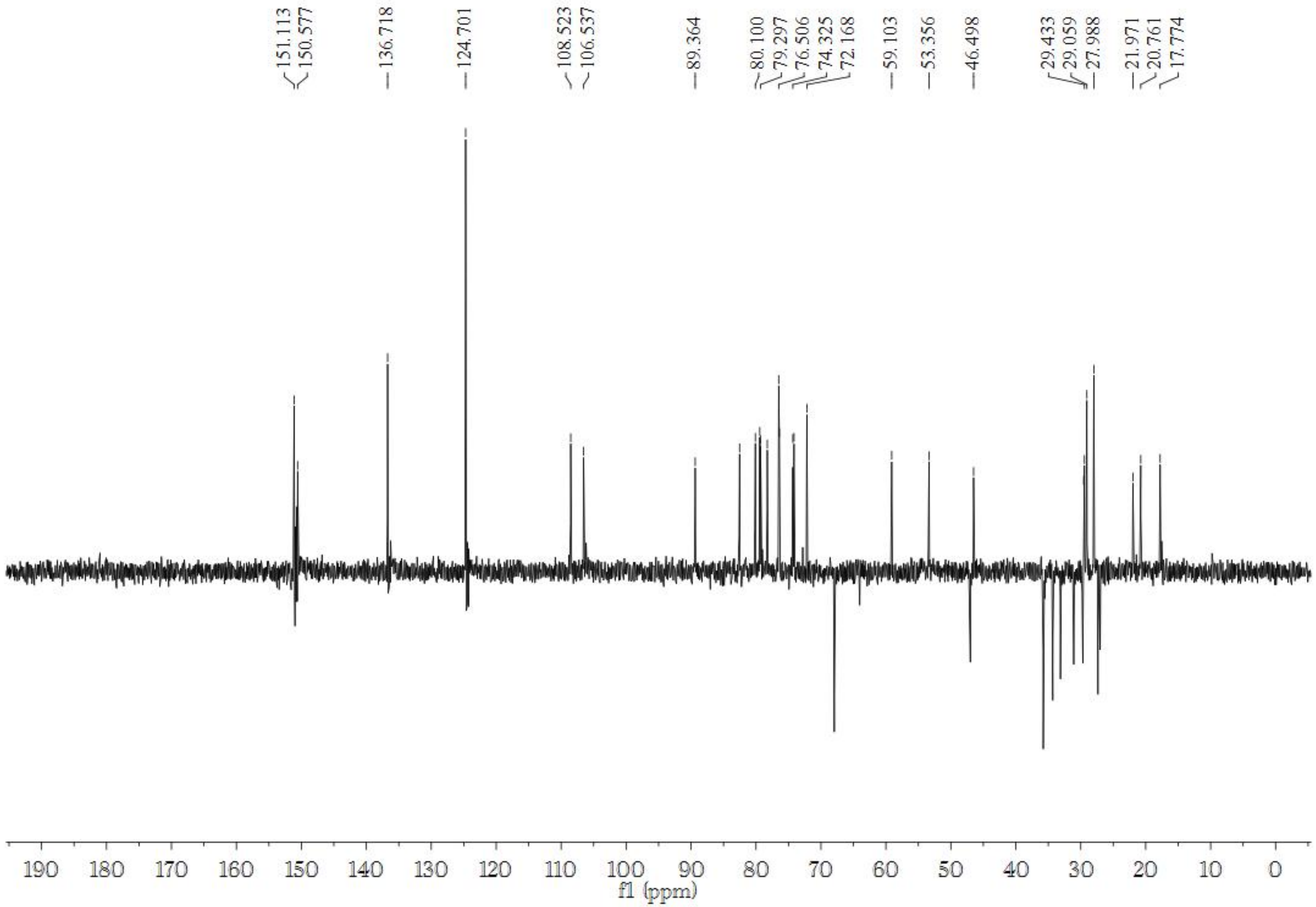
151.113
150.577
136.718
124.701
108.523
106.537
89.364
80.100
79.297
76.506
74.325
72.168
59.103
53.356
46.498
29.433
29.059
27.988
21.971
20.761
17.774
190 180 170 160 150 140 130 120 110 100 90 80 70 60 50 40 30 20 10 0
f1 (ppm)

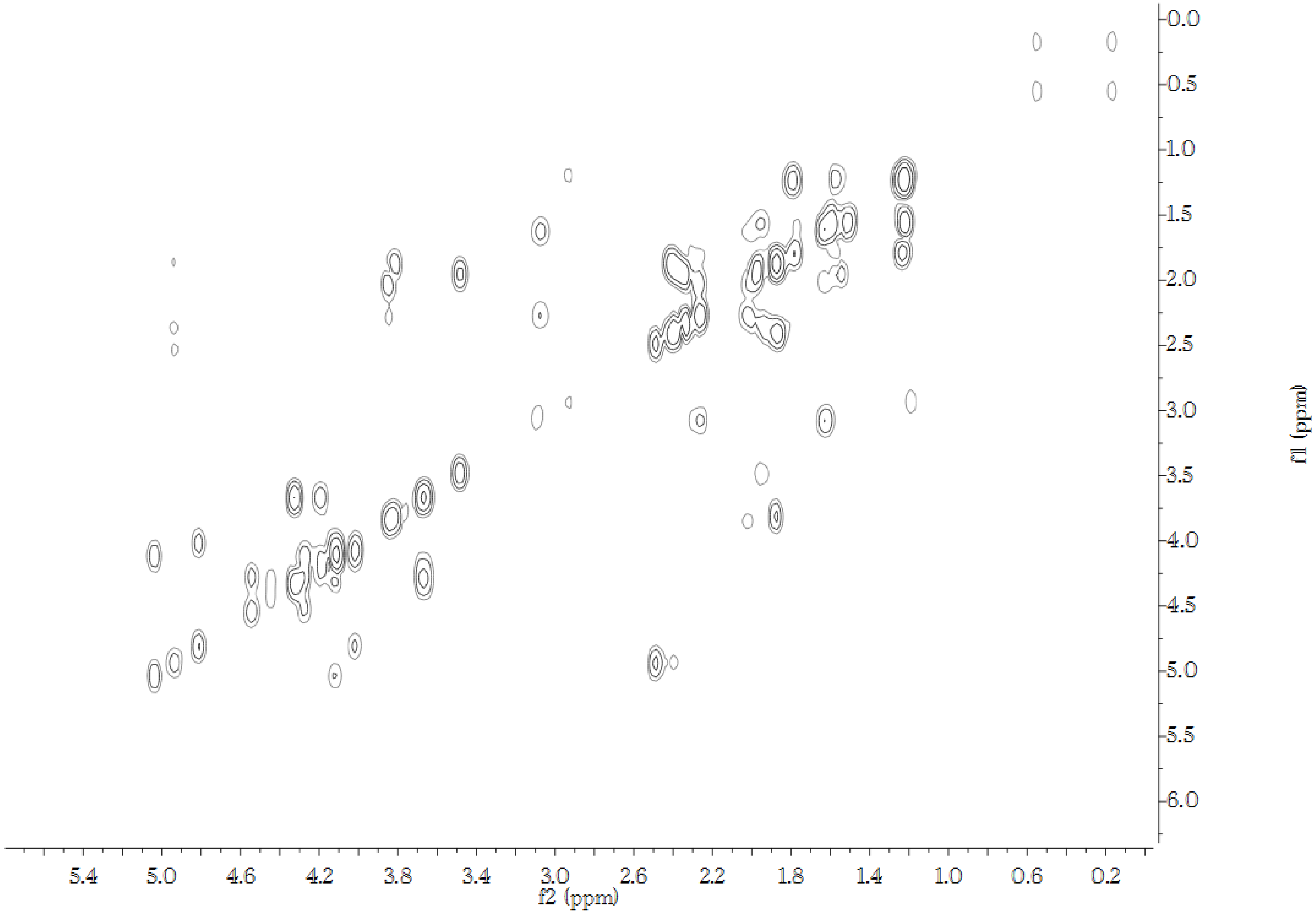

0.0
0.5
1.0
1.5
2.0
2.5
3.0
3.5
4.0
4.5
5.0
5.5
6.0
f1 (ppm)
5.4
5.0
4.6
4.2
3.8
3.4
3.0
2.6
2.2
1.8
1.4
1.0
0.6
0.2
f2 (ppm)

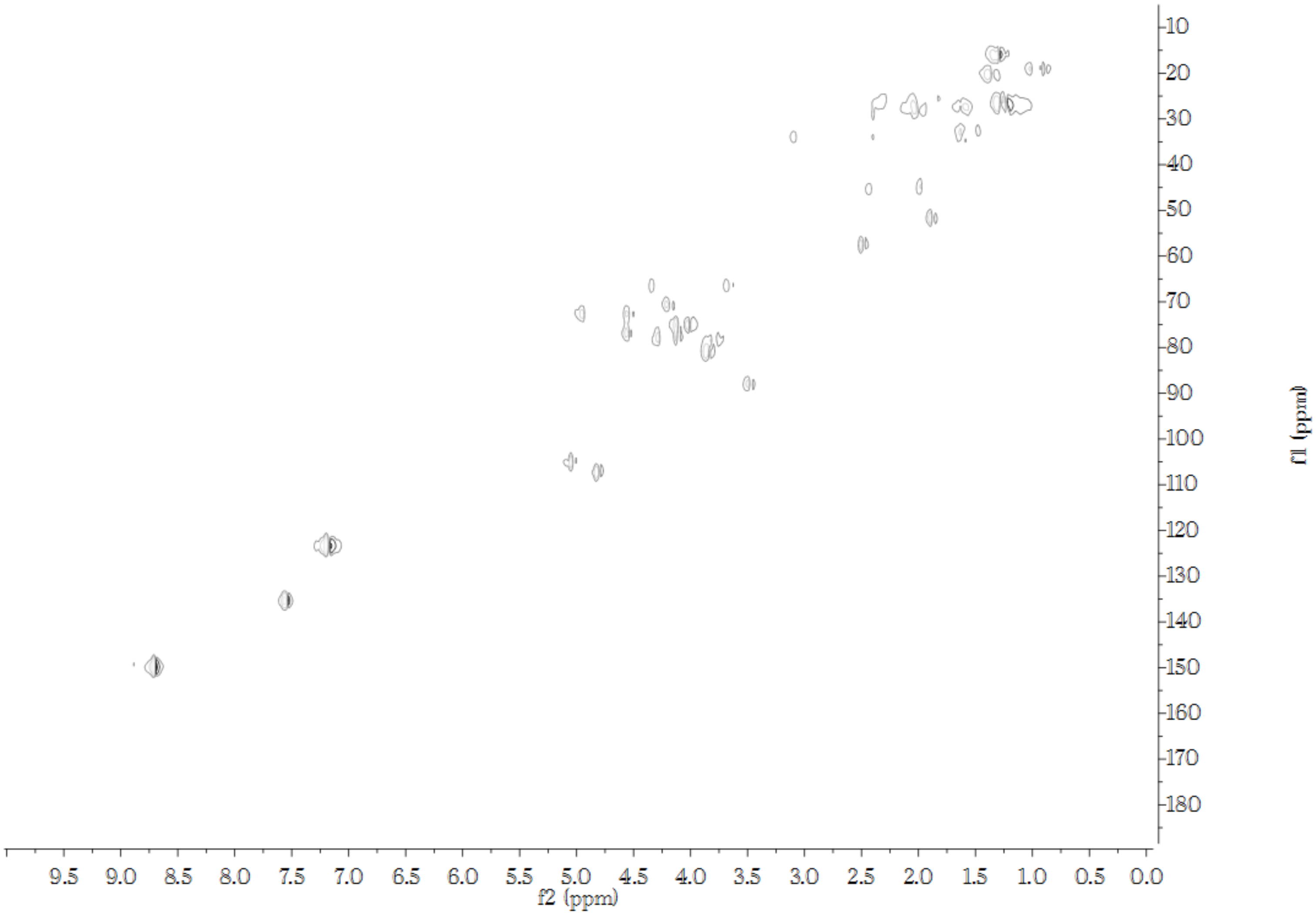

f2 (ppm)
f1 (ppm)

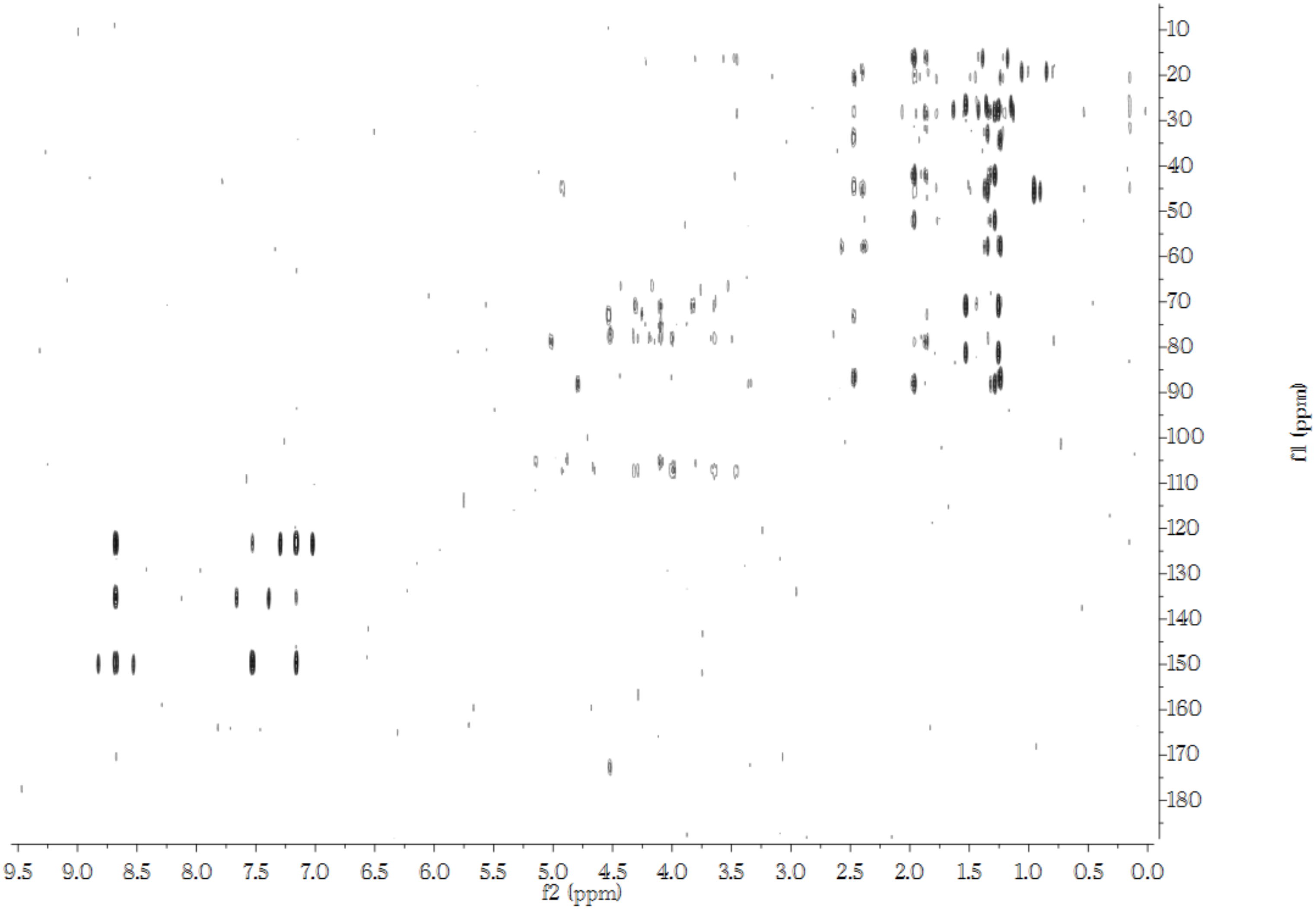

f1 (ppm)
f2 (ppm)
